# Enzymatic digestibility and ethanol fermentability of AFEX-treated starch-rich lignocellulosics such as corn silage and whole corn plant

**DOI:** 10.1186/1754-6834-3-12

**Published:** 2010-06-09

**Authors:** Qianjun Shao, Shishir PS Chundawat, Chandraraj Krishnan, Bryan Bals, Leonardo da Costa Sousa, Kurt D Thelen, Bruce E Dale, Venkatesh Balan

**Affiliations:** 1 Biomass Conversion Research Laboratory, Department of Chemical Engineering and Material Science, 3900 Collins Road, University Corporate Research Complex, Michigan State University, Lansing, MI 48910, USA; 2 School of Engineering, Zhejiang Forestry University, Linan, Zhejiang 311300, China; 3 Department of Crop and Soil Sciences, Michigan State University, East Lansing, MI 48824, USA; 4 DOE Great Lakes Bioenergy Research Center, Michigan State University, East Lansing, MI 48824, USA; 5 Department of Biotechnology, Indian Institute of Technology Madras, Chennai 600 036, India

## Abstract

**Background:**

Corn grain is an important renewable source for bioethanol production in the USA. Corn ethanol is currently produced by steam liquefaction of starch-rich grains followed by enzymatic saccharification and fermentation. Corn stover (the non-grain parts of the plant) is a potential feedstock to produce cellulosic ethanol in second-generation biorefineries. At present, corn grain is harvested by removing the grain from the living plant while leaving the stover behind on the field. Alternatively, whole corn plants can be harvested to cohydrolyze both starch and cellulose after a suitable thermochemical pretreatment to produce fermentable monomeric sugars. In this study, we used physiologically immature corn silage (CS) and matured whole corn plants (WCP) as feedstocks to produce ethanol using ammonia fiber expansion (AFEX) pretreatment followed by enzymatic hydrolysis (at low enzyme loadings) and cofermentation (for both glucose and xylose) using a cellulase-amylase-based cocktail and a recombinant *Saccharomyces cerevisiae *424A (LNH-ST) strain, respectively. The effect on hydrolysis yields of AFEX pretreatment conditions and a starch/cellulose-degrading enzyme addition sequence for both substrates was also studied.

**Results:**

AFEX-pretreated starch-rich substrates (for example, corn grain, soluble starch) had a 1.5-3-fold higher enzymatic hydrolysis yield compared with the untreated substrates. Sequential addition of cellulases after hydrolysis of starch within WCP resulted in 15-20% higher hydrolysis yield compared with simultaneous addition of hydrolytic enzymes. AFEX-pretreated CS gave 70% glucan conversion after 72 h of hydrolysis for 6% glucan loading (at 8 mg total enzyme loading per gram glucan). Microbial inoculation of CS before ensilation yielded a 10-15% lower glucose hydrolysis yield for the pretreated substrate, due to loss in starch content. Ethanol fermentation of AFEX-treated (at 6% w/w glucan loading) CS hydrolyzate (resulting in 28 g/L ethanol at 93% metabolic yield) and WCP (resulting in 30 g/L ethanol at 89% metabolic yield) is reported in this work.

**Conclusions:**

The current results indicate the feasibility of co-utilization of whole plants (that is, starchy grains plus cellulosic residues) using an ammonia-based (AFEX) pretreatment to increase bioethanol yield and reduce overall production cost.

## Background

Impending energy shortages and widespread environmental pollution are two major challenges facing humanity in the 21^st^ century. Petroleum is an important and scarce resource that meets 44% of the world's total energy demand. The increasing worldwide demand for crude oil and the dwindling petroleum resources have led to the development of alternative sources of fuel that can displace fossil fuels [1,2]. Many nations have initiated programs to develop alternative fuels, such as the 'Office of Energy Efficiency and Renewable Energy's Biomass Program', which aims to replace 20% of gasoline consumed in the USA, with alternative renewable fuels over the coming decade [3]. Ethanol is one such alternative renewable fuel that can potentially replace gasoline.

Currently, corn grain is the major US feedstock for producing fermentation-based ethanol, produced using either the wet or dry grind process[4]. Processes using starch or sucrose to produce ethanol are considered to be first-generation biorefineries. However, to sustainably scale up biofuel production, second-generation lignocellulosic biorefineries have been proposed to address the ongoing 'food versus fuel' argument, to meet the increasing demand for ethanol, and to reduce production costs [5-7]. Previously published work has demonstrated a significant improvement in lignocellulosic cell wall digestibility after ammonia fiber expansion (AFEX)-based pretreatment [8,9]. AFEX modifies grass lignocellulosic cell walls through decrystallization of cellulose, partial depolymerization of hemicellulose, and cleavage of ester-based lignin carbohydrate complexes (LCC) [10-12]. Ammonia can be recovered and reused during the process with no separate liquid stream being generated [8].

Conversion of starch-rich grain to ethanol involves wet thermal pretreatment to form starch slurries that are hydrolyzed by thermostable amylases to glucose, and then fermented to ethanol by native yeast strains [13]. By contrast, conversion of cellulose-rich corn stover to ethanol involves acidic or alkalinic thermochemical pretreatments, followed by enzymatic hydrolysis and fermentation by recombinant ethanologens such as *Saccharomyces cerevisiae *424A (LNH-ST) [14]. The energy consumed and resources utilized to convert corn grains and stover to ethanol through the two different processes described above could be minimized by developing a single-step process (that is, a whole-crop biorefinery) to simultaneously convert mature whole corn plants (WCP) or immature corn silage (CS) to ethanol [5,15,16].

CS is prepared by harvesting the whole plant (grain + stover) before physiological maturity, when the whole plant moisture level is approximately 60-70% (total weight basis; TWB). The harvested material is compacted to minimize exposure to oxygen and stored under moist conditions either in silos or in polythene bags for a period ranging from 20 to 200 days [17,18]. During this storage period, anaerobic microbes modify the substrate while growing on easily accessible carbohydrates. This leads to the production of a highly digestible animal feed with sufficient nutrients (such as, protein) from the microbes. As a result of lactic acid formation, the pH drops to 4, which preserves the silage from further microbial attack. At present, silage is used to feed ruminants, and is believed to be a potential feedstock for cellulosic ethanol-based biorefineries. It is widely believed that there would be significant cost savings from harvesting and processing WCP rather than separately processing grain and stover for production of biofuels [19].

In this paper, we demonstrate a 'one-pot' conversion of starch-rich grains and cellulosic stover to ethanol using CS- and WCP-based substrates via AFEX pretreatment, enzymatic hydrolysis and hydrolyzate fermentation by a recombinant *S. cerevisiae *424A (LNH-ST) strain.

## Results and Discussion

### Hydrolysis of AFEX-treated starch and cellulose

**Figure 1:**
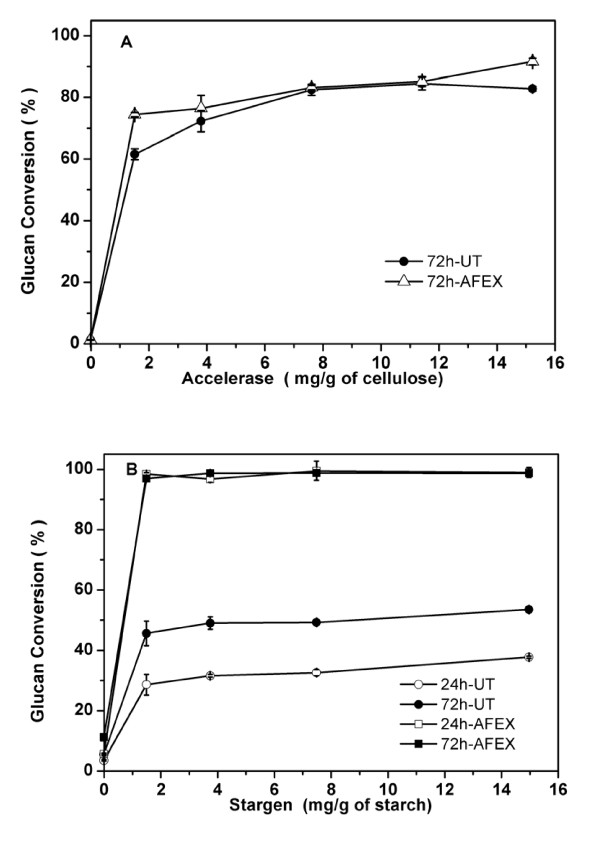
**Enzymatic hydrolysis of soluble starch and cellulose (Avicel), with or without ammonia fiber expansion (AFEX) treatment and as a function of different total protein loadings (as mg enzyme/gram glucan)**. Cellulose and starch hydrolysis carried out using **(a) **Accellerase and **(b) **Stargen.

CS and WCP contain a significant proportion of both starch and cellulose (Table 1) unlike typical lignocellulosics such as corn stover. Initial enzymatic hydrolysis studies were carried out using untreated and AFEX-treated cellulose (Avicel) and soluble starch to study the relative digestion kinetics as a function of enzyme loading. Percentage glucan conversion from cellulose and starch as a function of different protein loading (Accellerase™ and Stargen™) respectively, are shown in Figure 1. Avicel and starch were treated with AFEX under identical conditions as described in the Methods section. The glucose yield after hydrolysis of Avicel for 24 h (data not shown) and 72 h (Figure 1a) was largely comparable for untreated and AFEX-treated substrates for different protein loadings. These results are comparable with previous reports on the digestion kinetics of cellulose treated with ammonium hydroxide 28% w/w [20].

**Table 1:** Compositional analysis (dry weight basis) of corn silage (CS) with (1X-CS and 10X-CS) and without (0X-CS) ensilation, where 1 × represents addition of 0.0015 gm of inoculants (Silo-King) per gram of substrate for ensilation.

Components	Corn silage			Whole corn plant
		
	0X-CS	1X-CS	10X-CS	
Glucan (cellulose + starch)	49.2 ± 1.4	45.9 ± 0.7	44.8 ± 2.5	64.7 ± 0.6
Cellulose	19.7 ± 1.2	19.1 ± 0.2	19.6 ± 0.3	15.5 ± 0.4
Starch	29.5 ± 0.2	26.8 ± 0.5	25.2 ± 2.2	49.2 ± 1.0
Xylan	11.4 ± 0.2	12.1 ± 0.4	11.6 ± 0.2	12.2 ± 0.4
Arabinan	2.7 ± 0.1	2.9 ± 0.5	2.4 ± 0.1	1.9 ± 0.1
Klason lignin	8.8 ± 0.7	8.6 ± 0.5	8.9 ± 0.6	7.1 ± 0.1
Crude protein	10.2 ± 1.8	8.2 ± 0.4	8.9 ± 0.4	7.1 ± 0.5
Crude fat	3.9 ± 0.4	3.0 ± 0.6	3.3 ± 0.8	ND
Water-soluble carbohydrates	3.1 ± 0.3	4.4 ± 0.2	4.1 ± 0.1	5.1 ± 0.3
Ash	3.7 ± 0.3	4.1 ± 0.1	6.3 ± 0.1	2.1 ± 0.1

X = the manufacturer recommended loading rate of 0.0015 g/g.

For AFEX-treated starch, the glucose yield after 24 or 72 h of hydrolysis was 2.5-4-fold higher than that obtained from untreated starch (Fig 1b). Hot concentrated ammonium hydroxide is thought to gelatinize starch through disruption of inter- and intramolecular hydrogen bonding, similar to that seen during treatment of crystalline cellulose with liquid ammonia [21,22]. Disruption of the hydrogen bonds would create a more disordered ultrastructure and improve glucan chain hydration, greatly enhancing susceptibility to amylases. AFEX pretreatment and enzymatic hydrolysis of milled corn grain using Stargen™ (3 mg per gram starch) yielded 84% starch conversion within 12 h compared with 65% for untreated material (data not shown). However, after 72 h of hydrolysis, > 95% conversion was achieved for both substrates. The difference between the digestibility of soluble starch (typically isolated using acids) and corn grains may be due to differences in their ultrastructure.

**Figure 2:**
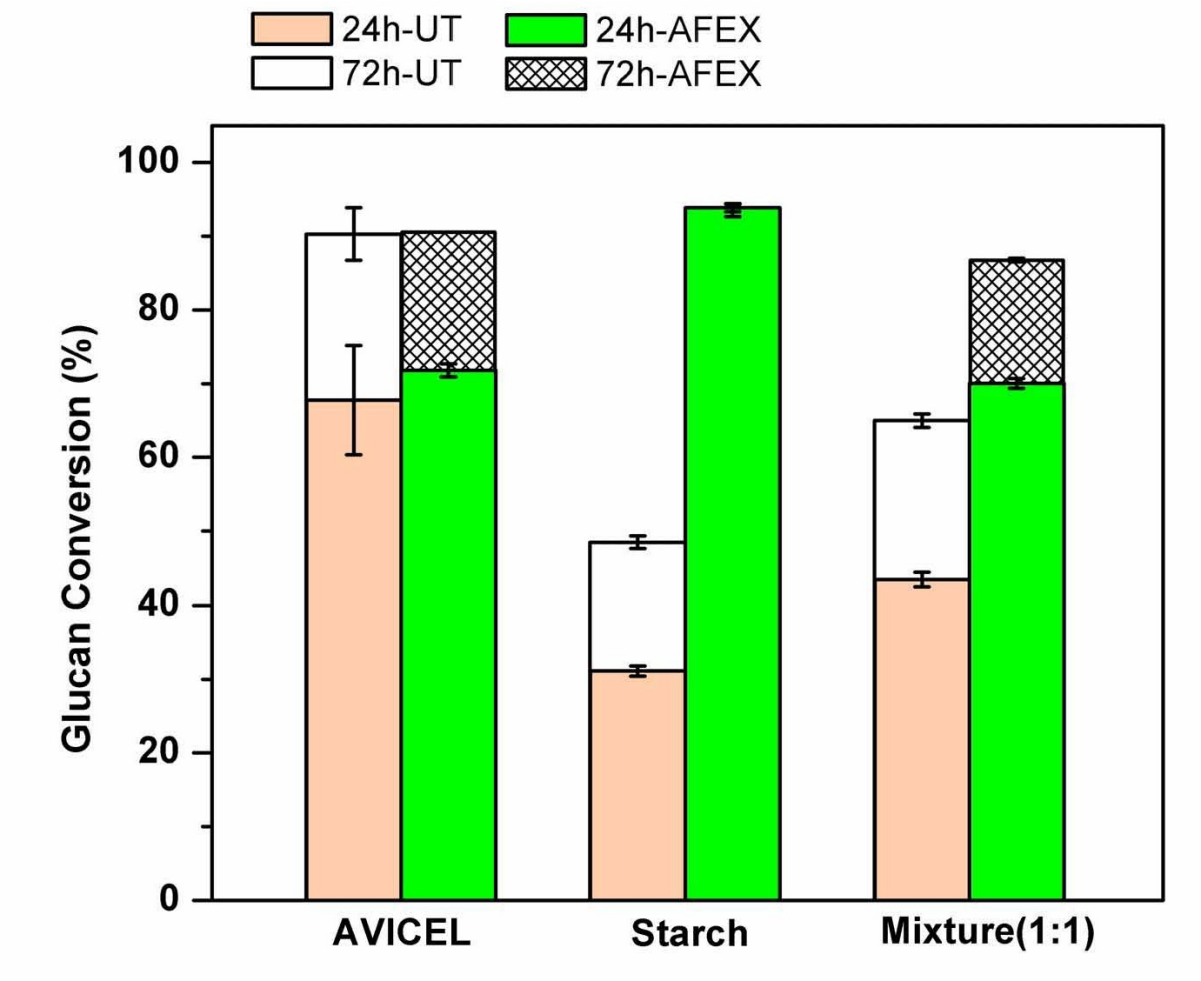
**Glucan conversion for untreated and ammonia fiber expansion (AFEX) -treated Avicel, starch and Avicel-starch mixture (1:1 w/w)**. Enzyme loadings for Avicel (Accellerase at 10.7 mg/g of glucan), starch (Stargen at 7.5 mg/g of glucan) and Avicel-starch mixture (Accellerase at 10.7 mg/g of cellulose and Stargen 7.5 mg/g of starch).

**Figure 3:**
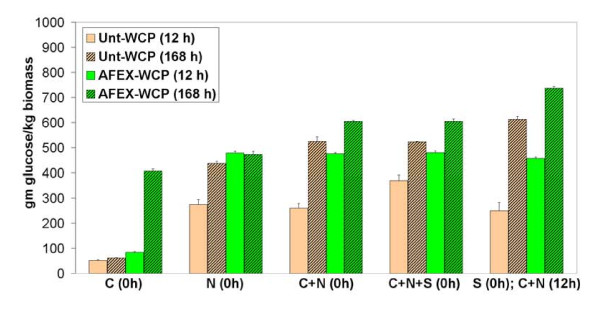
**Glucan conversion for untreated (yellow) and ammonia fiber expansion (AFEX)-treated (green) whole corn plant (WCP) (open bars, 12 h; striped bars, 168 h) for different loadings of commercial enzymes and hydrolysis conditions (time of enzyme addition)**. Spezyme CP (C) (23 mg/g of glucan), Novozyme 188 (N; 38 mg/g glucan) and Stargen (S) (5 mg/g glucan) were added as depicted on the X-axis at the respective time points (t = 0 or 12 h). The theoretical maximum possible glucose yield from WCP is 718 g glucose per kg dry weight biomass.

To mimic the hydrolysis of matured whole plant-based substrates, a mixture of Avicel and starch (with and without AFEX treatment) was hydrolyzed with a combination of Accellerase™ and Stargen™-based enzymes (Figure 2). The mixed substrate containing Avicel and starch at a ratio of 1:1 (dry weight basis; DWB) was pretreated with AFEX and hydrolyzed with a mixture of Accellerase™ (10.7 mg/g of cellulose) and Stargen™ (7.5 mg/g of starch). The AFEX-pretreated Avicel-starch mixture had higher glucan conversion compared with the untreated control. There was a 25-30% increase in glucan conversion after AFEX pretreatment. The maximum glucan conversion (at 72 h) of the AFEX-pretreated Avicel-starch mixture was essentially comparable with that of the independently hydrolyzed substrates. This result indicates that both starch and cellulose, within whole plants, can be pretreated using AFEX to maximize overall glucan yield. However, an interesting observation during these experiments was the marginal reduction in overall yields by 5-10% for the mixed substrate, suggesting that nonspecific binding of enzymes to cellulose and starch could result in slightly lower conversions. Further support for this hypothesis was found by conducting sequential hydrolysis of starch (by Stargen™) followed by cellulose (by Spezyme CP™) in WCP (Figure 3). Interestingly, the overall glucan conversions for both untreated and AFEX-treated WCP were 15-20% higher if amylases were added 12 h before addition of cellulases. Novozyme 188™ was also found to have significant amylase activity, giving close to 60-70% glucose yield within 12 h when used for hydrolysis of AFEX-treated WCP. It was also found that deactivating (by thermal denaturation) the amylases before the addition of cellulases resulted in marginally higher conversions (data not shown). Interestingly, hydrolysis of AFEX-treated WCP with Spezyme CP™ yielded higher glucose yield (> 400 g/kg biomass at 168 h) than that from untreated WCP (< 100 g/kg biomass). This suggests that trace amylase activity in Spezyme CP™ was able to digest AFEX-pretreated WCP more effectively than the untreated substrate during prolonged incubation (similar to that in Figure 1b comparing untreated and AFEX-treated starch). By contrast, the extent of glucan (both starch and cellulose) hydrolysis for both untreated and AFEX-treated WCP was comparable in the presence of only Novozyme 188™ (which is abundant in amylase activity; 5300 IU/mL [23]). These results suggest that in enzyme-limiting conditions, the beneficial role of pretreatment becomes much more evident. Spezyme CP™ is typically supplemented with Novozyme 188™ to prevent cellobiose inhibition due to lack of sufficient β-glucosidase activity in the former (unlike Accellerase 1000™, which has sufficient β-glucosidase activity). For the sake of simplicity and to help accentuate differences between untreated and AFEX-treated substrates, all further experiments were conducted using Accellerase 1000™ and Stargen™ added simultaneously (at time t_0_) at significantly lower protein loadings (typically < 10 mg/g starch or cellulose).

### Effect of AFEX pretreatment conditions on CS digestibility

**Figure 4:**
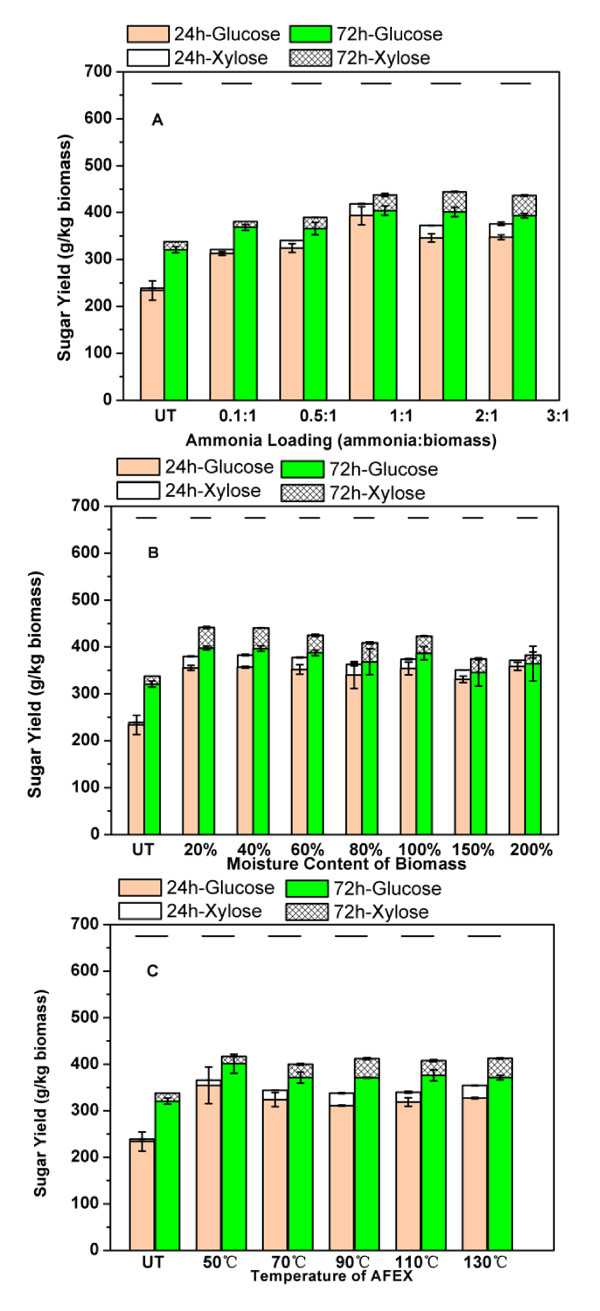
**Glucose and xylose released (g/kg dry weight biomass (DWB)) during hydrolysis of untreated (open bars) and ammonia fiber expansion (AFEX)-treated (striped bars) corn silage (0X-CS)-based substrates**. AFEX pretreatment was performed at different **(a) **ammonia to biomass loadings (w/w; DWB) at 90°C, 60% (DWB) moisture content, **(b) **moisture loadings at 90°C and 1:1 (w/w) ammonia to biomass loading and **(c) **temperatures at 60% (DWB) moisture loading and 1:1 (w/w) ammonia to biomass loading. Enzymatic hydrolysis was carried out at 50°C, 150 rpm in 15 mL reaction volume using Accellerase (3.1 mg/ g of cellulose) and Stargen (3.8 mg/g of starch). The lines above all bars indicate the maximum possible total glucose and xylose yield based on theoretical glucan and xylan composition.

The effects of AFEX pretreatment conditions on enzymatic digestibility of uninoculated CS (0X-CS) were explored. Ammonia loading was varied between 0.1 and 3 g per g biomass at 90°C, 60% moisture (DWB) and 5 min residence time. The glucan conversion increased with increased ammonia loading up to 1 g/g biomass (Figure 4a). Further increases in ammonia loading beyond this point did not significantly improve glucan hydrolysis. When moisture content was varied (20-200%, DWB) under similar conditions, maximum glucan conversion was achieved at lower moisture loadings (20%) (Figure 4b). At higher moisture levels, glucan conversion decreased marginally. Untreated CS that was dried (UT) before hydrolysis gave the lowest glucan conversion. However, it was found necessary to dry the samples (0X-CS) before AFEX pretreatment at lower moisture loadings. Temperature was varied between 50 and 130°C at fixed moisture content (60%), ammonia to biomass loading (1:1 w/w) and residence time (5 min). There was no major effect of temperature on glucan conversion within the range tested (Figure 4c). However, xylan conversion increased by 2-3-fold at temperatures > 90°C compared with 50°C. This is probably due to extensive cleavage of LCC ester complexes at higher temperatures, which enhances xylan digestibility [12]. In addition, there was less variability in glucan conversion at higher temperatures (90-130°C). Therefore, the AFEX conditions used for further experiments on dried CS (0X-CS, 1X-CS or 10X-CS) were 90°C, 1:1 ammonia loading, 60% moisture and 5 min residence time to maximize glucan and xylan conversions.

### Effect of microbial inoculation on pretreatment efficacy and enzymatic digestibility of ensiled corn plants (CS)

**Figure 5:**
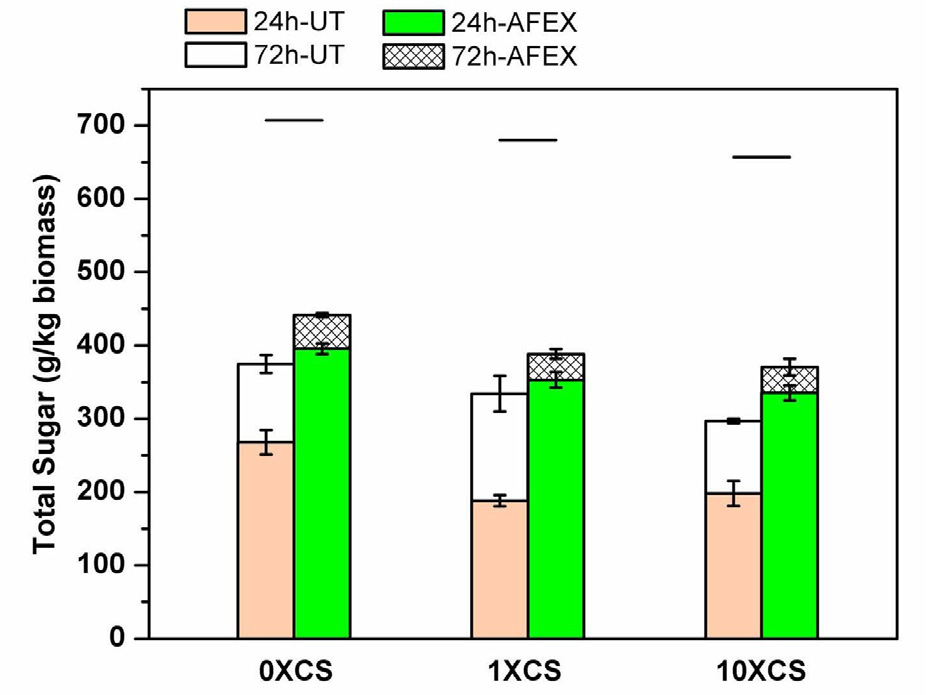
**Effect of ensilation (0X, 1X or 10X) of corn silage (CS) on enzymatic digestibility (total sugar = glucose + xylose + arabinose) before and after ammonia fiber expansion (AFEX) pretreatment**. AFEX conditions employed were as follows: temperature 90°C, moisture loading 60% DWB, ammonia to biomass loading 1:1 w/w, and residence time 5 mins. Enzymatic hydrolysis was carried out at 50°C, 150 rpm, 15 mL reaction volume in presence of Accellerase (3.1 mg/ g of cellulose) and Stargen (3.8 mg/g of starch). The lines above all bars indicate the maximum possible total sugar yield based on theoretical composition.

The effect of microbial inoculation on the composition and glucan digestibility of CS (before and after AFEX pretreatment) was studied. Although the cellulose and hemicellulose contents were not altered significantly, starch content was reduced by 10-15% upon inoculation of CS (Table 1). AFEX pretreatment of dried CS samples (with/without inoculation) resulted in 15% lower hydrolysis yields for 10X-CS compared with 0X-CS (Figure 5). These results indicate that microbial inoculation before the ensiling process was not beneficial to overall glucan hydrolysis yields. Previous work has shown that biological pretreatment of lignocellulosic cell walls with fungi (for example, white rot *Pleurotus ostreatus) *before conventional thermochemical processing enhances enzymatic digestibility of cellulose by cleaving LCC linkages [24]. The mechanisms by which lignin restricts cell wall degradation and hydrolysis of cellulose are not well understood. Enteric fermentation studies suggest that the influence of lignin on microbial deconstruction of plant cell walls may be primarily due to physical mechanisms such as shielding of cellulose but the effect of lignin may also involve more specific molecular interactions [25,26]. However, the microbes (for example, *Lactobacillus, Pediococcus*) present during conventional ensilation lack the enzymatic activities required to cleave LCC linkages and improve overall substrate digestibility.

### High solid loading-based CS and WCP enzymatic hydrolysis

**Figure 6:**
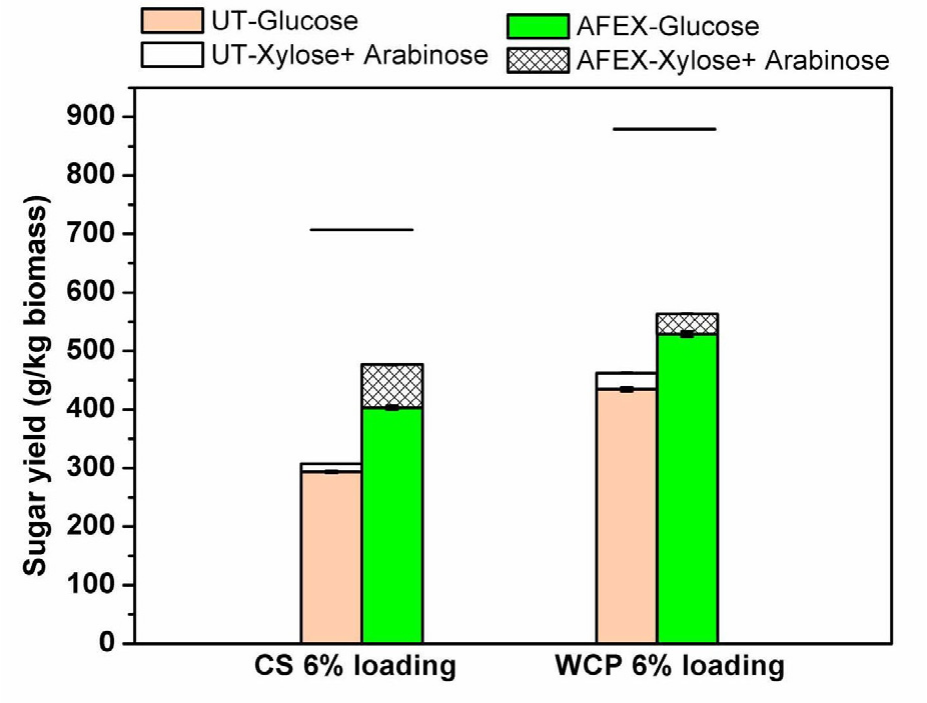
**Glucose and xylose yields during high-solid (6% w/w glucan loading) loading-based enzymatic hydrolysis for corn silage (0X-CS) or whole corn plant (WCP) before and after ammonia fiber expansion (AFEX) treatment**. AFEX conditions employed were as follows: temperature 90°C, moisture loading 60%, DWB, ammonia to biomass loading 1:1 w/w, and residence time 5 min. Enzymatic hydrolysis was carried out at 50°C, 72 h, 250 rpm, 500 mL reaction volumes in the presence of Accellerase (10 mg/g of cellulose), Stargen (3.50 mg/g starch) and Multifect Xylanase (7.5 mg/g Xylan). The lines above all bars indicate the maximum possible sugar yield based on theoretical composition.

Untreated and AFEX-treated (90°C, 1:1 ammonia loading, 60% moisture and 5 min residence time) CS (0X-CS) samples were hydrolyzed under high solid loading (that is, 6% w/w glucan loading) conditions to increase sugar concentration in the hydrolyzate and maximize ethanol titer (Figure 6). To further increase xylose yield, the cellulase (Accellerase™) and amylase (Stargen™) cocktails were supplemented with hemicellulases (Multifect Xylanase) during high-solid loading-based hydrolysis. As expected, AFEX pretreatment of silage significantly enhanced both glucan and xylan conversions. The glucose and xylose yields from AFEX-treated silage were 1.8- and 4.4-fold higher than untreated silage (at 1% glucan loading; data not shown). The glucan conversion was 70% and was about 10% lower at 6% glucan loading than at 1% glucan loading, because of the higher concentration of monomeric sugars in the hydrolyzate, which are known to inhibit cellulase activity [27,28]. The enzymatic digestibility of WCP at 6% glucan loading was also tested before and after AFEX pretreatment. However, it was found that the overall hydrolysis yield at 6% glucan loading for AFEX-treated WCP was significantly lower that that reported previously (Figure 3). This is probably due to the significantly lower protein loading we used (10-15-fold difference), compounded by end-product inhibition of hydrolytic enzymes at 6% glucan loading compared with previously reported experiments.

### Ethanol fermentation of CS- and WCP-based hydrolyzates

**Figure 7:**
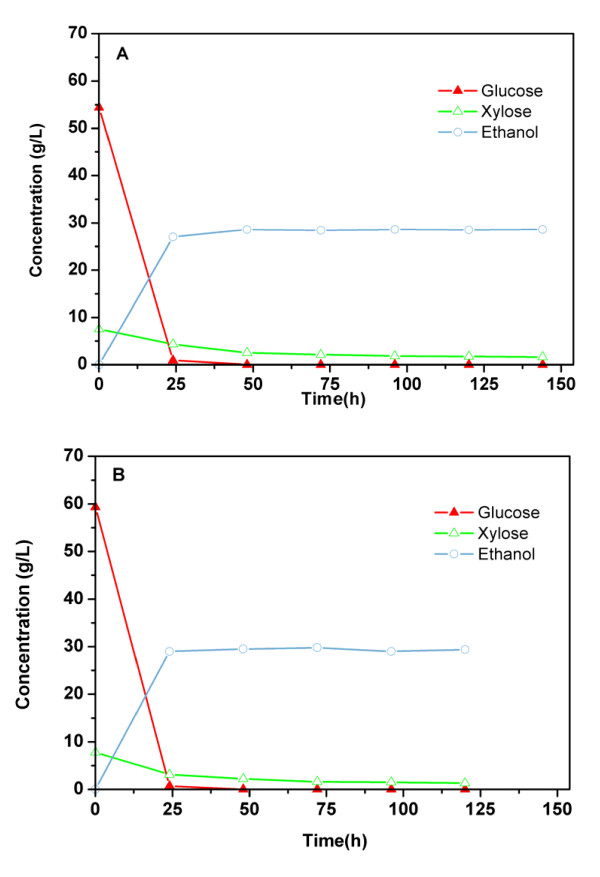
**Ethanol production and sugar utilization by *S. cerevisiae *424A for enzymatic hydrolyzates of ammonia fiber expansion (AFEX) -treated (a) corn silage (0X-CS) and (b) whole corn plant (WCP). Y-axis depicts glucose, xylose and ethanol concentrations in g/L**.

High solid loading-based enzymatic hydrolyzates of untreated and AFEX-pretreated 0X-CS and WCP were used for ethanol fermentation. Figure 7a depicts the consumption of glucose and xylose during production of ethanol from the 6% glucan loading-based AFEX-treated 0X-CS hydrolyzates. The hydrolyzate of untreated silage contained 45.4 g/L of total sugar (94.1% glucose and 5.9% xylose; data not shown). Total sugar concentration increased to 62.0 g/L (87.8% glucose and 12.2% xylose; Figure 7a) during hydrolysis of AFEX-pretreated 0X-CS. During fermentation of AFEX 0X-CS hydrolyzate by *S. cerevisiae *424A (LNH-ST), glucose was almost completely consumed within 24 h, but, about 27% of the xylose remained unused even after 72 h (Figure 7). Ethanol concentration in the broth was 28 g/L after 72 h, which corresponds to 93% theoretical yield based on total sugar consumption. In untreated silage, 23 g/L ethanol with a metabolic yield of 98% was produced at 72 h (data not shown).

Enzymatic hydrolysis of AFEX-treated WCP at 6% glucan loading resulted in a yield of 59 g/L glucose and 7.5 g/L xylose. The profile of the sugars consumed and ethanol produced during fermentation of hydrolyzate of AFEX-pretreated WCP is shown in Figure 7b. Glucose utilization was almost complete in 24 h, and xylose utilization was 80-82% at 72 h. The overall ethanol concentration was slightly higher for WCP (30 g/L) than for 0X-CS, but the metabolic yield of ethanol was slightly lower (86-89%) from WCP than from CS (93%) (Table 2). The different availability of nitrogenous nutrients and inhibitory organic acids could be responsible for differences in the metabolic yield of ethanol during fermentation [14]. Based on the glucan content, AFEX pretreatment efficiency and metabolic yield of ethanol fermentation, the calculated yield of ethanol from AFEX-treated CS was 69.3 gallon/ton of biomass, which was 1.5-fold higher than that of untreated silage (45.5 gallon/ton). AFEX-treated WCP gave slightly better ethanol yield than AFEX-treated CS.

**Table 2:** Ethanol fermentation of hydrolyzates (at 6% w/w glucan loading) of AFEX-pretreated whole plants using recombinant *S. cerevisiae *424A.

Sample	Glucose, g/L		Xylose, g/L		Ethanol, g/L	Metabolic yield, %	Productivity, g/L/h
				
	0 h	72 h	0 h	72 h			
0X-CS	54.4	0	7.5	2.1	28.4	93.2	0.39
WCP	59.3	0	7.8	1.6	29.8	89.2	0.41

CS, corn silage; WCP, whole corn plant.

X = the manufacturer recommended loading rate of 0.0015 g/g

## Conclusion

AFEX was shown to be an effective pretreatment for enhancing enzymatic digestibility and fermentability of starch-rich lignocellulosics such as CS and WCP (among other whole-grain crops such as wheat and rice; data not shown). The goal of the current study was to maximize fermentation titer and minimize biorefinery processing costs through cohydrolysis (at low enzyme loadings; < 10 mg total protein/g glucan) of both starch- and cellulose-based feedstocks for producing biofuels. It was found that sequential enzymatic hydrolysis of cellulose and starch yielded 15-20% higher yields, suggesting a possible antagonistic interaction between amylases and cellulases on a complex starchy cellulosic substrate. Microbial inoculation of corn plants before AFEX pretreatment did not benefit glucose hydrolysis yield, essentially due to loss of starch during ensilation.

Co-utilization of starch-rich grains and lignocellulosic residue for production of biobased commodity chemicals has a number of economic benefits. However, future commercialization of this process would require significant changes in harvesting practices and on-field equipment, development of biomass storage and transportation options, optimization of starch- and cellulose-based thermochemical co-pretreatment, and minimization of enzyme loadings required for efficient hydrolysis.

## Materials and methods

### Biomass, chemicals and enzymes

Corn plants, either in the immature state to be used for ensiling (CS) or as mature WCP were obtained from Michigan State University Farms (East Lansing, MI, USA). The corn hybrid used was NK 49-E3 (Syngenta, Basel, Switzerland) which is a typical CS hybrid used in the Great Lakes Region. The corn plants used in this study were planted on 8 May 2008 and harvested on 19 September 2008 for ensilation. The WCP were harvested after the plant reached physiological maturity, which occurred approximately 6 weeks after harvest for ensilation. WCP was harvested as stover and grain separately (moisture content < 15% DWB). WCP-based samples were milled using a Wiley mill (Christy and Morris, Chelmsford, UK) (10 mm sieve attachment) followed by mixing of the grain and stover fractions at a mass ratio of 1:1 (w/w). Ensiling was accomplished by sealing 500 g immature entire corn plant samples in plastic bags using a commercial grade vacuum seal food machine (CG-15; Cabela, Sidney, NE, USA). The sealed bags were stored at 21°C for 30 days to imitate a typical on-farm ensiling process. We also evaluated the effect of a commercially available microbial inoculant product (Silo-King, Agri-King Inc., Fulton, IL, USA) on ensiled corn digestibility at a 0X, 1X and 10X loading (X = the manufacturer recommended loading rate of 0.0015 g/g). The inoculant product is composed of lactic acid-producing organisms, such as *Lactobacillus plantarum, Pediococcus pentosaceus *and *Enterococcus faecium*, and is used by farmers to enhance the feed quality of ensiled corn [29]. The CS samples were frozen using liquid nitrogen, milled using a laboratory blender (Hamilton Beach, Washington, NC, USA), and passed through a 10 mm screen sieve. The milled CS samples were stored in sealed Ziploc Storage Bags (SC Johnson, Racine, Wisconsin, USA) at -20°C for long-term storage. The moisture content of CS was between 63 and 67% (DWB). The CS samples were dried to < 10% moisture (DWB) using a 50°C oven, to allow suitable adjustment of the water loadings used during pretreatment.

Avicel PH101 and soluble starch S5160599 (lot #054261) were purchased from Fluka (Tokyo, Japan) and Fisher Scientific (USA), respectively. Commercial enzymes used for degrading cellulose were Spezyme CP™ (88 mg/ml) and Accellerase 1000™ (84 mg/ml; lot #1600844643) (both gifts from Genencor Division, Danisco US Inc., Rochester, NY, USA), The enzymes used for degrading starch were Novozyme 188™ (149 mg/ml) (Sigma-Aldrich (Sigma, St. Louis, MO, USA) and Stargen 001™ (62 mg/ml; lot #4900851951) (gift from Genencor Division). The enzyme used for degrading hemicellulose was Multifect Xylanase™ (35 mg/ml) (gift from Genencor Division). The concentrations of these enzymes were estimated using a Kjeldahl-based method (Dairy One Feed Stock Analyzing Co., Ithaca, NJ, USA).

### Compositional analysis

Crude protein, starch, crude fat and water-soluble carbohydrate content of CS (0X, 1X and 10X) and WCP were determined at the Forage Testing Laboratory (Dairy One Inc.). In addition, acid and neutral detergent fiber values were determined for WCP. Polysaccharide (cellulose, xylan and arabinan), Klason lignin, extractive and ash content were determined based on the standard National Renewable Energy Laboratory protocols [30]. Glucan content refers to total cellulose and starch composition of the substrate. WCP was composed of 49.2% starch and 15.5% cellulose (total glucan 64.7%).

### AFEX pretreatment

AFEX pretreatment was carried out as described previously [11]. After charging liquid ammonia into the reactor containing the biomass at the appropriate moisture content, the reactor temperature was raised rapidly to the desired level and held constant for 5 min. Subsequently, ammonia was rapidly released through the exhaust valve. The treated biomass was removed from the reactor and air-dried overnight in a fume hood to remove residual ammonia. AFEX was carried out on CS at different moisture loadings (20 to 200% DWB), temperatures (50°C to 130°C) and ammonia loadings (0.1-3 g ammonia per gram dry weight of biomass). WCP, starch and Avicel samples were pretreated with AFEX at 90°C for 5 min reaction time (total residence time in the reactor after injection of ammonia was ~ 25-30 min), 60% moisture (DWB) and 1:1 (w/w) ammonia to biomass loading.

### Enzymatic hydrolysis

AFEX-treated substrates were used without washing with water before hydrolysis. Enzymatic hydrolysis of substrates was carried out based on the National Renewable Energy Laboratory (NREL) protocol [30] at a total volume of 15 ml using screw-capped vials. The substrate was hydrolyzed in 50 mM sodium citrate buffer (pH 4.8) at various enzyme loadings (as mg protein per gram cellulose, starch or xylan). Tetracycline (40 mg/L) and cycloheximide (30 mg/L) were added to prevent microbial growth. Hydrolysis was conducted at 50°C with mild agitation (150 rpm). Sampling was carried out at 12, 24, 72 and 168 h.

### High solid loading-based enzymatic hydrolysis

High solid loading hydrolysis was based on 6% glucan (cellulose + starch) loading for each substrate. The pretreated substrate was hydrolyzed in fed-batch mode in two stages (3% glucan loading for each stage) separated by a 24 h time interval. The hydrolysis was carried out in a 2000 ml conical flask (500 ml reaction volume) with 50 mM sodium citrate buffer (pH 4.8) and incubated at 50°C with shaking at 250 rpm. After 24 h, a second batch of solids and appropriate quantity of enzymes were added to the flasks and incubated under identical conditions for an additional 48 h. Tetracycline at 40 mg/L was added to avoid microbial growth during hydrolysis. The hydrolyzates were centrifuged at 10,000 rpm (10, 100 × g) for 30 min, and the supernatants were sterilized by filtration for subsequent ethanol fermentation.

### Analytical methods

Separation and quantification of monomeric sugars was conducted using a high performance liquid chromatography (HPLC) machine equipped with an automatic sampler ( LC2010; Shimadzu Scientific Instruments, Columbia, MD, USA) and refractive index detector (Waters RI Detector, 410; Waters Corporation, Milford, MA, USA). For acidic-based hydrolyzates, a HPX-87H Aminex column (Bio-Rad, Hercules, CA, USA) maintained at 65°C using a 5 mM sulfuric acid-based mobile phase (flow rate of 0.6 mL/min) was used for monosaccharide analysis, and a HPX-87P Aminex column maintained at 85°C using water as the mobile phase (0.6 ml/min) was used for analysis of enzymatic hydrolyzates. The concentrations of glucose, xylose and ethanol in the fermentation broths were simultaneously estimated using the HPX-87H column.

### Fermentation culture and media

Genetically engineered *S. cerevisiae *424A (LNH-ST) was obtained from Dr Nancy Ho (Purdue University, West Lafayette, IN, USA). This strain contains xylose-metabolizing genes integrated into the host chromosome [31]. This strain was cultured routinely in YEPX (1% yeast extract, 2% peptone and 2% xylose) medium at 30°C with shaking at 150 rpm. The culture was maintained on YEPX-agar plates at 4°C for regular use.

### Ethanol fermentation

The seed culture was prepared by inoculating YEP-glucose medium with cells from the plate culture followed by incubation at 30°C with agitation at 150 rpm. After 48 h, the cells were harvested by centrifugation. The supernatant was discarded and cells were transferred to 100 ml of fresh fermentation medium in 250 ml Erlenmeyer flasks. The flasks were closed with rubber stoppers pierced with a thin surgical needle to allow release of the carbon dioxide formed during fermentation. The inoculated flasks were incubated at 30°C with agitation (100 rpm) in a temperature-controlled orbital shaker. The culture growth was monitored by measurement of optical density at 600 nm. The initial OD_600 _of all cultures was about 0.1. During fermentation, 1 ml culture samples were removed at regular time intervals and analyzed for glucose, xylose and ethanol. The metabolic ethanol yield (Y_p/s_) was calculated as the mass of ethanol produced per unit mass of sugar utilized during fermentation. The theoretical yield of ethanol for glucose or xylose is 0.51 g ethanol per gram sugar. Volumetric ethanol productivity (Q_v_) of fermentation was calculated as the amount of ethanol (g/L) produced per unit time (h) of fermentation.

## Competing interests

The authors declare that they have no competing interests.

## Authors' contributions

QS carried out the experiments on corn silage and drafted the manuscript, SC performed the experimental work on WCP and helped write the manuscript, LS conducted the high solid loading-based hydrolysis, and CK carried out the subsequent fermentations. KT conceived the corn silage idea and provided the samples. BB carried out experimental work on whole grains. VB and BD helped draft the manuscript and coordinated the overall study. All authors read and approved the final manuscript.
